# Public’s early response to the novel coronavirus–infected pneumonia

**DOI:** 10.1080/22221751.2020.1732232

**Published:** 2020-03-03

**Authors:** Siyi Zhan, Ying Ying Yang, Chuanxi Fu

**Affiliations:** From the School of Public Health, Zhejiang Chinese Medical University, Hangzhou, People’s Republic of China

To the editor: Originated from Wuhan city in central China and widely spread due to the mass migration for Lunar new year holiday, 17,000+ confirmed novel coronavirus (2019-nCoV)-infected pneumonia (NCIP) cases with 350+ deaths have been identified in the country since December 2019 [1–2]. Another 100+ exported cases have been confirmed in other countries worldwide [3]. During 10:30am to 10:30pm, January 29th, 2020 (UTC + 8), we initiated an online survey on people’s knowledge on NCIP by snowball sampling via Wechat invitation. 3083 invitees of 18–59 years old (2/3 females and 4/5 with bachelor’s degree or higher) from all provinces in mainland China were enrolled, three quarters of which were from the six most developed provinces in China (Zhejiang, Guangdong, Shandong, Beijing, Shanghai, Jiangsu).

Worried on the highly contagious virus that currently lacks effective treatments, 84.9% (83.6–86.2%) subjects (2619) felt “extremely” or “very” nervous about NCIP, and 75.1% (73.6–76.6%) believed that it was at least as terrible as the Severe Acute Respiratory Syndromes (SARS) or avian influenza. Adults living in urban areas had a better awareness of the knowledge on NCIP than those in rural areas (72.7% vs.66.1%, *p* < 0.001). Such knowledge includes route of transmission (respiratory: 98.1% vs. 97.6%; contact: 95.4% vs. 91.5%), length of quarantine (97.2% vs. 95.1%), preventable vaccine (95.0% vs. 92.0%), drugs for treatment (84.7% vs. 77.9%), and time for mask replacement (89.0% vs. 86.6%). This finding indicates that Health education should be strengthened among adults living in rural areas where access to medical services is limited.

Protective measures such as washing hands, wearing masks and exercising can effectively prevent people from getting infected [4–5]. 87.8% (CI:86.6%–89.0%) subjects chose five or six items among all six measures provided. However, 36.3% (34.6%–38.0%) of subjects who would wear mask cannot wear it correctly, with common mistakes of nose exposure to the air and long-time mask wear without replacement. Males (female vs. male, OR: 0.544, 95% CI: 0.440–0.673), younger adults (1.844, 1.466–2.320), and subjects with higher education (2.200, 1.780–2.718) were associated with better behaviours. Our study indicates that general public in China has high concerns on the NCIP in its rapid development phrase of the outbreak. Among all subjects, 85.9% (84.7–87.1%) believed that the plague could be controlled within 3 months due to the stringent measures advocated by government. As the NCIP outbreak continues, further understanding of the infection will help to adjust strategies for public’ health education.
